# Interplay effect evaluation on lung and esophageal cancer patients treated with compact proton system in free breathing

**DOI:** 10.1016/j.phro.2026.101085

**Published:** 2026-09-17

**Authors:** Giorgio Cartechini, Esther Kneepkens, Gloria Vilches-Freixas, Richard Canters, Mirko Unipan, Marije Velders, Indra Lubken, Maud Cobben, Anouk Vullings, Janou Buck, Maud van den Bosch, Steffy Paulissen, Julie Schoemakers, Dianne Hartgerink, Judith van Loon, Stephanie Peeters, Ilaria Rinaldi

**Affiliations:** Maastro, Maastricht Radiation Oncology Institute, Maastricht, the Netherlands

**Keywords:** Interplay effect, Proton therapy, Motion management, Organ motion, Mevion S250i

## Abstract

**Background and purpose:**

Pencil beam scanning proton therapy is sensitive to respiratory motion, which can cause dose discrepancies due to the interplay between tumor and beam delivery motion. This study evaluates the clinical impact of interplay for lung and esophageal cancer patients treated in free-breathing, without rescanning.

**Materials and methods:**

Interplay was evaluated in 36 patients (18 lung, 18 esophageal) using both predictive and *a posteriori* approaches. The predictive method, for pre-treatment assessment, employed 24 synthetic sinusoidal breathing patterns to model respiratory motion. The *a posteriori* method incorporated patient-specific breathing traces and machine log files. Fraction-accumulated dynamic 4D dose distributions were generated and compared with the institutional 4D robustness evaluation.

**Results:**

The predictive method agreed closely with *a posteriori* evaluation, with a median Relative Distribution Error < 1% across all dose-volume metrics. Interplay-induced underdosage in the first fraction (−1% to −2%) decreased after ∼5 fractions, with cumulative D_98%_, D_95%_ and V_95%_ values converging to within 2% of planned doses. Two plans, one lung and one esophageal, showed persistent underdosage exceeding 5%, consistent with 4D robustness evaluation failures. For the esophageal case, a revised beam arrangement restored robustness.

**Conclusions:**

Internal target volume-based robust optimization combined with machine-specific beam delivery characteristics provides motion robustness for most lung and esophageal patients treated in free-breathing. Additional motion mitigation was not required for most patients treated under conventional fractionation. However, small targets with large motion remain at risk of interplay-induced underdosage, warranting caution, as this combination may compromise plan robustness.

## Highlights

• ITV-based 3D robust optimization yielded motion-robust plans.
• Interplay underdosage (∼1–2%) converged within 5 fractions.
• Small, high-motion targets were more sensitive to intra-fraction motion.
• Predictive and *a posteriori* evaluations agreed, with median relative error < 1%.

## Graphical abstract

**Figure:**
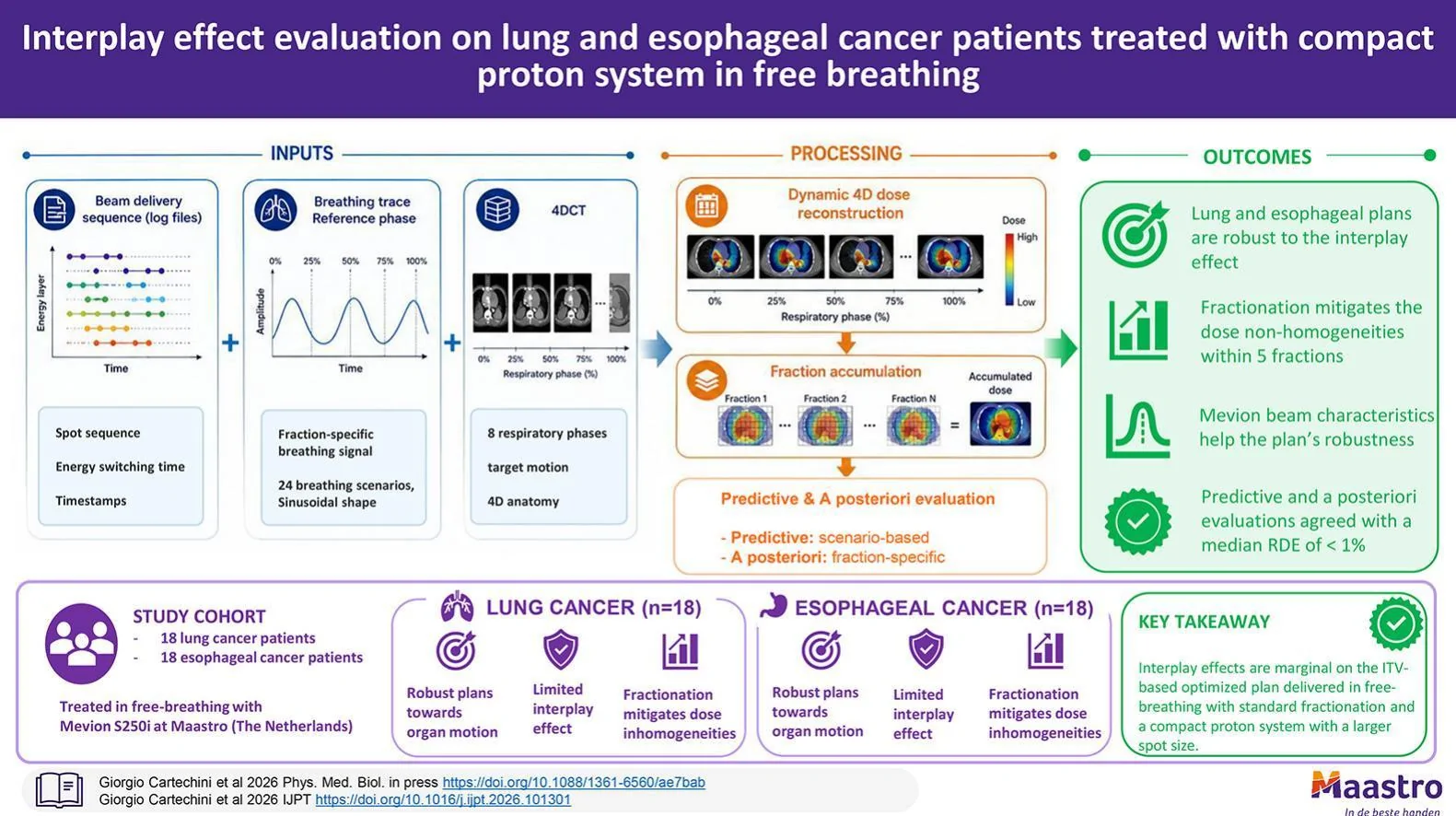

## 1 Introduction

Proton radiotherapy using pencil beam scanning (PBS) presents significant challenges when treating moving targets. Intra-fractional tumor motion and beam delivery occur on comparable timescales, potentially causing discrepancies between spot delivery and tumor position. This phenomenon, known as the interplay effect, can lead to reduced target coverage within a single fraction and an increased dose to surrounding organs of interest (OOI) [1], [2], [3], [4]. The magnitude of this effect depends on multiple factors, including machine-specific delivery parameters, patient-specific breathing patterns, and treatment plan characteristics [5], [6].

To address this challenge, various real-time respiratory motion management strategies have been proposed and implemented. A recent interinstitutional survey by Zhang et al. [7] investigated the status of these techniques. Globally, 85% of clinical respondents reported using rescanning [8], [9] or active motion mitigation strategies such as beam gating, often combined with breath-hold techniques [10], [11], [12], [13]. However, each of these techniques prolongs the treatment time. Therefore, pre-treatment strategies have been investigated to either evaluate or optimize the robustness of the plan against organ motion, as well as range and setup variations. Both 3D and 4D robust optimization have been shown to produce clinically acceptable plans in terms of robustness of target coverage and OOI sparing against the motion effects [14], [15], [16], [17]. According to the Particle Therapy Co-Operative Group (PTCOG) Thoracic and Lymphoma Subcommittee consensus guideline and the AAPM Task Group Report 290, a retrospective study on institutional patients should support the deployment of motion mitigation strategies [18], [19]. To support this, a wide range of robust evaluation methods have been developed to assess plan quality with respect to intra- and inter-fractional organ motion after optimization [20], [21]. At our institution, Maastro Clinic (The Netherlands), lung and esophageal cancer patients are treated using the Mevion S250i proton system under free-breathing conditions without rescanning. This system is characterized by a larger spot size compared with other PBS systems, which may improve plan robustness against organ motion [22]. To ensure plan robustness, each plan is 3D robustly optimized on the Internal Target Volume (ITV), and a 4D robustness evaluation (4DRE) has been developed and routinely employed to assess the plan's robustness against organ motion, range, and setup variations [23]. The main limitation of the 4DRE is that it does not account for the beam delivery sequence and, therefore, the interplay effect.

In a previous study, we presented predictive and *a posteriori* methods to assess the interplay effect as well as range and setup errors [24]. This work systematically evaluates the impact of the interplay effect on the lung and esophageal treatments at our institution and assesses the robustness of our predictive interplay method in a larger patient cohort. We assessed whether the current treatment strategy, combined with the Mevion S250i machine characteristics, requires additional motion mitigation strategies to be implemented.

## 2 Materials and methods

### 2.1 Beam delivery characteristics

All treatments were delivered with the Mevion S250i Hyperscan system, which accelerates protons to a fixed energy of approximately 227 MeV and uses 18 range-shifter plates to generate 161 clinically available energy layers. The uncollimated in-air spot size at isocenter, measured with a 10 cm air gap and expressed as one standard deviation, ranged from 4.1 mm at 227 MeV to 16.4 mm at 45 MeV [22], approximately twice that of other proton systems [25], [26].

From a temporal perspective, the inter-pulse time between consecutive spots has a broad distribution. It is characterized by a short spot drilling time of a few milliseconds, as well as longer delays associated with Adaptive Aperture motion [27]. The average energy layer switching time of this system is 50 ms [22], shorter than other proton systems: approximately 300 ms for the Varian ProBeam and around 1 s for the IBA ProteusPlus [28].

### 2.2 Interplay evaluation

To evaluate the robustness of treatment plans against organ motion, we employed the interplay evaluation framework developed by Cartechini et al. [24]. Both *a posteriori* and predictive methods were used to calculate the dynamically accumulated 4D dose (D4DD or dynamic dose) [18], leveraging the temporal resolution provided by 4DCT imaging. The 4DCT comprised eight respiratory phases: 0%, 25%, 50%, 75%, and 100% inhalation, as well as 25%, 50%, and 75% exhalation.

For both approaches, D4DD was calculated using a standard method [29], [30], [31]: beam spots were assigned to 4DCT phases based on delivery sequences from machine log files and respiratory traces. Doses were mapped to a reference phase (50% exhale) using deformable image registration (DIR) in RayStation 2024B (RaySearch Laboratories, Stockholm, Sweden).

The *a posteriori* approach used fraction-specific respiratory traces acquired *via* the Catalyst PT surface guidance system (C-RAD, Uppsala, Sweden), while the predictive approach employed 24 synthetic breathing curves based on a sinusoidal model with varying periods (2, 3.5, and 5 s) and initial phases (aligned with the eight 4DCT phases); these parameters were selected to represent the observed patient breathing patterns.

We used the predictive approach as a pre-treatment estimate of interplay effects, and the *a posteriori* method to validate the predictive model.

Agreement between the two methods was quantified using the Relative Distribution Error (RDE), defined as the average relative difference between percentile vectors derived from distributions of the same dosimetric quantity [29]. The effect of fractionation was evaluated by accumulating dose over the treatment course. For the predictive method, one of the 24 synthetic breathing scenarios was randomly selected per fraction, repeated 24 times to account for sampling variability, and the mean cumulative DVH metrics were analyzed as a function of fraction number. For the *a posteriori* method, cumulative dynamic dose was obtained from fraction-specific machine log files and respiratory traces [24].

Results were compared with the clinically implemented 4DRE method developed at our institution [23]. It exploits the 4D dose (4DD) [18], calculated as the weighted average of doses on all 4DCT phases accumulated on the reference phase. The 4DRE evaluates target coverage using voxel-wise minimum (VWmin) dose distributions [32]. The VWmin represents the minimum dose value distribution per voxel from all scenarios. For lung cases, VWmin D_95%_ to the CTVp must exceed 95% of the prescribed dose (Dp), while for esophageal cases, VWmin V_94%_ must cover at least 98% of the CTVp. The 4DRE is a simplified and faster approach to test the 4D plan robustness in clinical routine. However, it does not account for random uncertainties generated by the interplay between organ motion and beam delivery sequence.

### 2.3 Patient data

We recalculated D4DD for 36 patients (18 lung, 18 esophageal), comprising 45 treatment plans (nominal A-plans and adapted B-plans). In each cohort, eight patients were evaluated with both *a posteriori* and predictive approaches, while the remaining ten were assessed retrospectively using only the predictive method. Patients were selected to represent the largest motion scenarios: lung patients based on large Gross Target Volume (GTV) center-of-mass displacement across 4DCT phases, and esophageal patients based on diaphragm motion, defined as the maximum displacement between lower-lobe apexes. Unlike primary lung tumors, which are typically confined to a localized region of the lung, esophageal tumors can extend longitudinally from the clavicular region to the diaphragm. As a result, respiratory motion does not affect the entire esophageal target uniformly; regions closer to the diaphragm are more strongly influenced by breathing motion than superior regions. Therefore, diaphragm motion amplitude was used as a proxy for target motion in esophageal cancer patients.

Table 1, Table 2 summarize the characteristics of the lung and esophageal cohorts, respectively. All treatment plans were generated using 3D robust optimization on the ITV, defined as the union of the CTVp delineated across all 4DCT phases. Optimization consisted of 21 scenarios combining 3% range and 5 mm setup variations. The average CT was used as the planning reference image. To account for delineation uncertainties, the ITV was expanded by either 1 mm (lung) or 3 mm (esophagus). All nodes are treated as one structure, called CTVn, and no expansion of this structure is applied. In this study, we evaluate only the robustness of the CTVp.

**Table 1:** Lung cancer patients' characteristics. Nominal A-plans or adapted B-plans were delivered with either 3 or 2 beam angles. When specified, the ‘Twin beams’ (Tb) technique is used. The fractionation scheme is provided in Gy (RBE = 1.1) and number of treatment fractions (fr#).

Lung cancer patients						
ID	Fields	Plan	Fractionation scheme [Gy x fr#]	CTV Volume [cm^**3**^]	GTV Amplitude [mm]	*A posteriori* breathing traces
L1	3	A	1.5 × 30	87	9.7	Yes
L2	3	A	2 × 30	81	5.1	Yes
L2	3	B	2 × 30	75	13	Yes
L3	2 Tb	A	2.75 × 20	155	9.3	Yes
L3	2 Tb	B	2.75 × 20	148	10.9	Yes
L4	3 Tb	A	2.75 × 20	176	7.2	Yes
L4	3 Tb	B	2.75 × 20	118	7.2	Yes
L5	3	A	2 × 30	151	6.4	Yes
L6	3	A	2.75 × 20	33	9.0	Yes
L7	2 Tb	A	2.75 × 20	115	5.1	Yes
L8	2 Tb	A	2.75 × 20	16	10.7	Yes
L9	3	A	2 × 30	262	22.8	No
L10	3	A	2 × 30	262	5.0	No
L11	3	A	2 × 30	239	7.2	No
L12	3	A	2 × 30	50	5.1	No
L13	3	A	2 × 30	202	6.6	No
L14	3	A	2 × 30	223	14.9	No
L15	3	A	2.75 × 20	82	5.1	No
L16	3	A	2.75 × 20	58	12.3	No
L17	3	A	2.75 × 20	152	9.7	No
L18	3	A	2 × 30	242	12.6	No

**Table 2:** Esophageal cancer patients' characteristics. All the plans were delivered with three beam angles per isocenter. The fractionation scheme is provided in Gy (RBE = 1.1) and number of treatment fractions (fr#).

Esophageal cancer patients						
ID	Fields	Plan	Fractionation scheme [Gy x fr#]	CTV Volume [cm^**3**^]	Diaphragm Amplitude [mm]	*A posteriori* Breathing traces
O1	3	A	1.8 × 23	291	8.5	Yes
O2	3	A	1.8 × 23	232	8.9	Yes
O2	3	B	1.8 × 23	213	13.2	Yes
O3	3	A	1.8 × 23	181	22.5	Yes
O4	3	A	1.8 × 28	281	14.6	Yes
O5	6	A	1.8 × 23	489	11.5	Yes
O5	6	B	1.8 × 23	500	13.4	Yes
O6	3	A	1.8 × 23	219	9.1	Yes
O6	3	B	1.8 × 23	197	15.1	Yes
O7	3	A	1.8 × 23	156	20.1	Yes
O7	3	B	1.8 × 23	163	50.6	Yes
O8	3	A	1.8 × 28	179	30.3	Yes
O8	3	B	1.8 × 28	174	20.9	Yes
O9	3	A	1.8 × 23	99.7	19.4	No
O10	3	A	1.8 × 28	150	25.9	No
O11	6	A	1.8 × 23	508	36.8	No
O12	3	A	1.8 × 23	140	17.0	No
O13	3	A	1.8 × 28	199	21.8	No
O14	3	A	1.8 × 23	259	12.5	No
O15	3	A	1.8 × 23	58	39.7	No
O15	3	B	1.8 × 23	58	39.7	No
O16	3	A	1.8 × 23	510	21.4	No
O17	3	A	1.8 × 28	263	9.1	No
O18	3	A	1.8 × 28	527	12.7	No

Lung plans used two or three coplanar beam directions selected according to tumor location and organ-at-risk sparing criteria, as described by Taasti et al. [33]. In some cases, a ‘twin beams’ strategy was used [33], consisting of paired beams with different spot spacing but otherwise identical parameters to improve robustness without adding extra beam directions.

For the esophageal patients, plans were optimized with three coplanar beams or six beams with two isocenters for extended target regions. Further details regarding the treatment planning strategies and institutional protocols are provided in reference [34].

The Maastro Institutional Review Board approved and supervised the project (P0719).

## 3 Results

For the subset of patients for whom the breathing traces were recorded, we compared the *a posteriori* and predictive approaches by calculating the RDE parameter. Fig. 1 shows the distribution of RDE for the D_98%_, D_95%_ and V_95%_ of the CTVp for both lung and esophageal cancer patients. The median RDE for both lung and esophageal cancer patients was below 1% for the CTVp for all the DVH parameters. Only one lung cancer patient showed high RDE values above 2.5%, indicating a large difference in DVH value distributions between *a posteriori* and predictive methods. This plan was delivered in only three treatment fractions, and the limited *a posteriori* data were insufficient to represent the distribution of dose metrics.

**Fig. 1:**
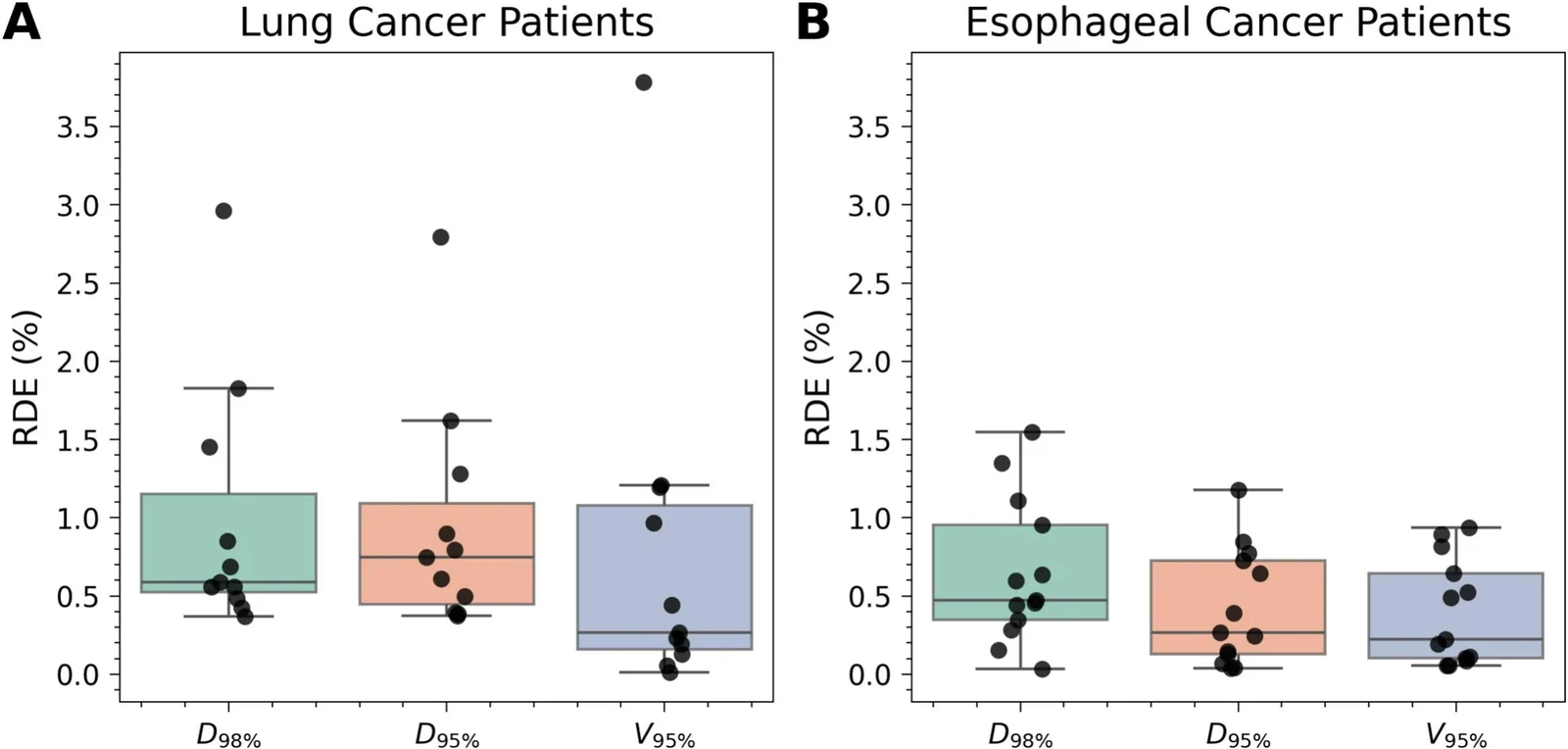
Relative distribution error (RDE) distribution as a function of DVH parameters for CTVp on lung (A) and esophageal cancer patients (B).

Fig. 2(A-C) shows the relative percentage differences between cumulative DVH parameters and the nominal CTVp plan values over the prescribed treatment fractions for the lung cancer cohort. Each box plot represents the inter-patient distribution of the mean accumulated D4DD DVH metrics derived from the predictive interplay evaluation. For all plans, we report the mean D4DD DVH metrics obtained from the predictive interplay evaluation.

**Fig. 2:**
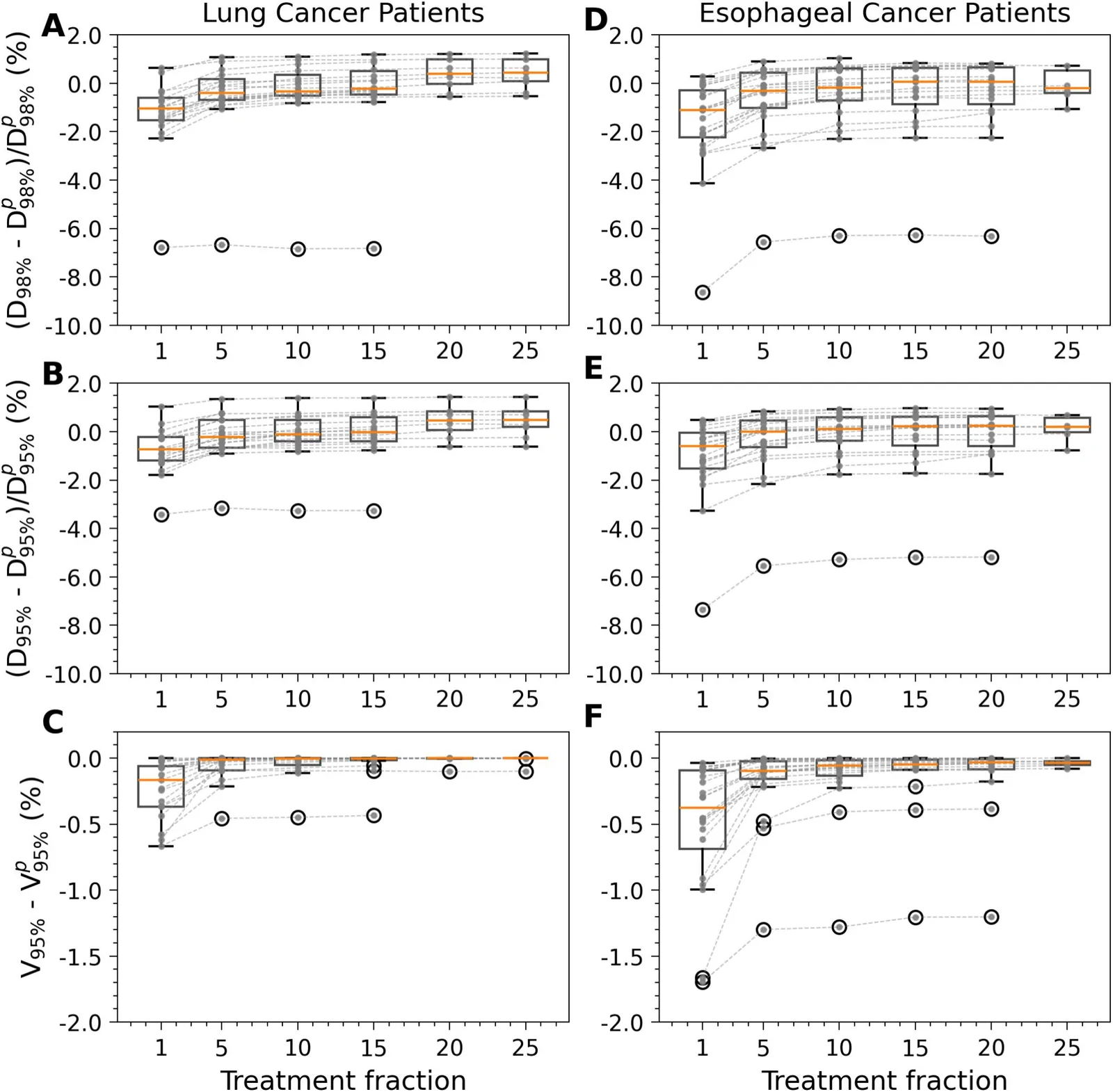
The relative difference between D_98%_, D_95%_, and V_95%_, and the planning values (D^p^_98%_, D^p^_95%_, and V^p^_95%p_) for CTVp of lung (A-C) and esophageal (D-F) patients. Each boxplot represents the inter-patient distribution of the DVH parameters averaged over the simulated treatment courses. The dashed grey line connects the points of the same patient over the treatment fractions. The circles indicate outliers in the box-plot distributions.

A consistent trend is observed across all panels. For the CTVp, deviations from the prescribed dose during the first fraction ranged between −1% and − 2%. However, all DVH metrics converged within 1% of the planned values after approximately five fractions. Fig. 2(C) shows a rapid narrowing in the spread of V_95%_ values over the treatment course, with convergence to a 0% relative difference for nearly all patients. One patient exhibited notably larger deviations (L8). The D_98%_, D_95%_, and V_95%_ values for L8 are shown as outliers in the inter-patient distribution.

A similar analysis was conducted for the esophageal cancer cohort, as shown in Fig. 2(D-F). As with the lung cohort, DVH values generally converged toward the nominal plan values over the treatment course. After five fractions, D_98%_, D_95%_, and V_95%_ values were within 2% of the planned values. However, one plan (O15 A-plan) demonstrated persistent discrepancies throughout the treatment, with D_98__%_ and D_95%_ remaining more than 5% below the prescribed values.

The 4DRE confirmed the results shown in Fig. 2: all plans passed the robustness criteria except for L8 and O15 A-plan (Table S2 in Supplementary materials).

In our patient cohort, two cases showed large deviations from the planning values over the treatment course: one lung cancer patient (L8) and one esophageal cancer patient (O15). Fig. 3 illustrates the relative differences between the accumulated D4DD and the planned DVH parameters over all treatment fractions.

**Fig. 3:**
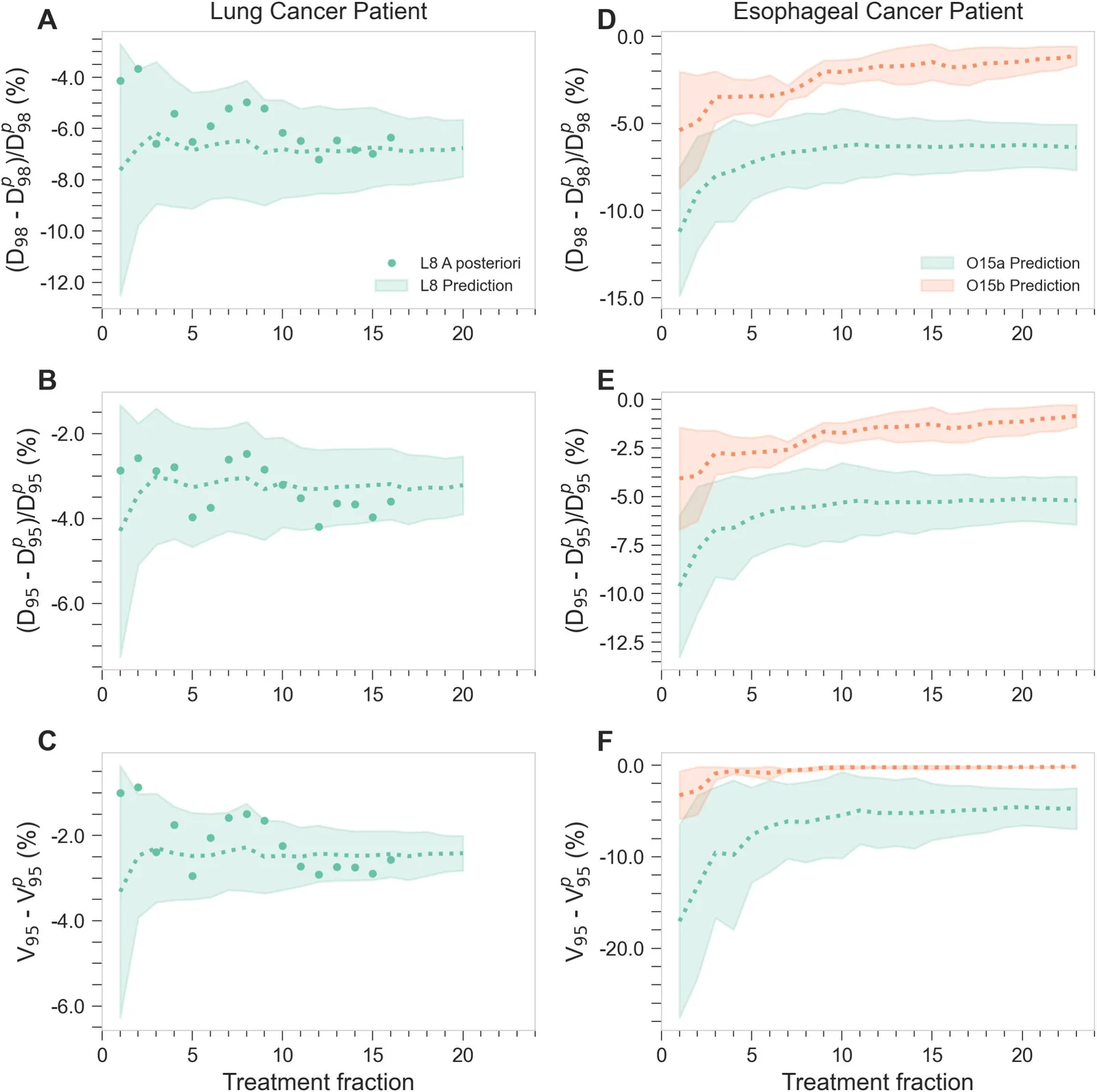
Panels display the relative difference between D_98%_, D_95%_, and V_95%_, and the planning values for the L8 lung patient (A-C) and the O15 esophageal patient (D-F) of the CTVp. The color band represents the standard deviation of the predictive method computed over 10 synthetic treatment courses, while the dashed line represents the average value. The points in panels A-C show the *a posteriori* data.

For the lung case (Fig. 3A-C), both the *a posteriori* and predictive evaluations showed good overall agreement. The DVH metrics for the CTVp revealed substantial deviations from the planned values, with D_98%_ underdosage exceeding 5%, and more than 2% deviation in both D_95%_ and V_95%_. The stable mean values observed in the predictive approach indicate that fractionation had a minimal impact on reducing the target dose inhomogeneities. However, fractionation contributes to the narrowing of the color band in the predictive curves. The 4DRE supported these results: the VWmin D_95%_ fell below the 95% prescribed dose threshold, which was defined as the clinical goal (see section A.3 of the Supplementary Materials).

For the esophageal case (Fig. 3C-F), both treatment plans A and B were analyzed. The large DVH deviations in the A-plan highlighted its lack of robustness against organ motion. Unlike the lung case, fractionation provided a modest improvement in target coverage during the first five fractions, most notably in V_95%_, which initially showed a deviation greater than 10% that gradually reduced to within 5%. The A-plan was delivered for the first two fractions, after which a new beam arrangement (B-plan) was introduced (see Fig. S2 of section A.2 of the Supplementary Materials) [35]. The new beam arrangement is characterized by one oblique posterior-anterior beam, one posterior-anterior beam, and one posterior-anterior tilted beam. Compared to the A-plan, the non-coplanar beam arrangement uses two beams that do not cross the diaphragm.

Following this change, both D_98%_ and D_95%_ converged within 2% of the prescribed dose after 10 fractions. The robustness improvement is also confirmed by the 4DRE: the VWmin V_94%_ of the B-plan met the acceptance criteria, being above 98% of the prescribed dose (see Table S2).

Patients L8 and O15 had the smallest CTV volumes in this study, measuring 16 cm^3^ and 58 cm^3^, respectively (Table 1, Table 2).

## 4 Discussion

In this study, we evaluated interplay effects in 36 patients, comprising 45 PBS treatment plans delivered under free-breathing without rescanning. Our results show that ITV-based robust optimization, following the conservative Dutch consensus [32], [36], produces clinically acceptable plans for both indications. This robustness likely reflects both the institutional planning approach and the delivery characteristics of the Mevion S250i system, including relatively large spot sizes and rapid delivery. Larger spot sizes have been reported to reduce sensitivity to interplay by smoothing motion-induced dose heterogeneities [5], [22], although this benefit is specific to motion robustness and it may come at the expense of reduced lateral conformity.

The strong agreement between *a posteriori* and predictive approaches (Fig. 1) confirms earlier findings [24]. The 24 sinusoidal breathing scenarios adequately approximated patient-specific respiratory patterns. Only one patient showed relatively high RDE values, reflecting greater disagreement between the methods; however, this case involved only three delivered fractions before adaptation, limiting the representativeness of the *a posteriori* data. Although the *a posteriori* interplay evaluation was used as the reference, it has limitations: it does not account for anatomical changes during treatment, and it assumes alignment between the patient-specific breathing trace and the 4DCT phase at treatment start [24].

Only two plans showed clinically relevant motion-induced target underdosage (Fig. 2, Fig. 3). These corresponded to the smallest CTVs, 16 cm^3^ (patient L8) and 58 cm^3^ (patient O15), suggesting that small targets with large motion are particularly vulnerable to loss of motion robustness. This is consistent with Kardar et al., who reported up to 16% CTV coverage differences between 4DD and D4DD in similar cases [37]. In both cases, 4DRE also failed (Tables S1 and S2), indicating that the observed underdosage was not primarily due to random interplay alone, but to a systematic motion-induced loss of target coverage. Fractionation reduced the variability caused by interplay, as shown by narrowing uncertainty bands in Fig. 3, but could not compensate for this underlying systematic deficit.

The O15 case further supports this interpretation: the adapted B-plan improved robustness through alternative beam angle selection, reducing diaphragm interaction and aligning beams with motion. This highlights the importance of patient-specific beam configuration, as also supported by 10.13039/100007325AAPM TG-290, which recommends water-equivalent path length analysis for robust beam selection [18], [38].

For all other plans, fractionation effectively reduced dose inhomogeneities within the first five fractions, confirming its role in averaging out respiratory motion and delivery-related effects [39], [40]. Although the physical effect of fractionation is equivalent to rescanning, the clinical relevance of possible fractional target underdosage remains unknown. Bortfeld and Paganetti showed that fractional dose variations below 10% marginally affect the normalized total dose, a general definition of the biological equivalent dose, independently of the biological parameters [41]. This was always the case for the patients analyzed in this work. These findings suggest that current exclusion criteria for lung cancer patients, GTV amplitude greater than 2 cm, could potentially be reconsidered. This motion threshold for lung patients was defined at our institution after a preliminary internal evaluation of plan robustness. This observation is supported by the re-evaluation of three lung patients previously treated with photons (Section A.4), as well as patient L9, all of whom demonstrated robust target coverage despite large motion amplitudes.

The interplay analysis and clinical 4DRE were fully consistent in this cohort, with no false positives or false negatives for plans showing relevant DVH deviations (Tables S1 and S2). This agreement is expected, as accumulated D4DD converges to 4DD with repeated fractions [42].

By incorporating delivery timing and respiratory dynamics, the interplay framework extends 4DRE and enables the assessment of both random interplay effects and systematic underdosage. In contrast, 4DRE offers a simpler and faster clinical workflow (168 *vs.* 192 dose calculations) and does not require machine log files. However, the predictive approach provides additional insight into temporal dose evolution and fractionation-driven mitigation.

This study has several limitations. Anatomical changes and inter-fractional motion variability were not explicitly modeled. In clinical practice, predictive evaluation is performed before repeated 4DCTs are available, but it can be repeated during adaptation. Previous studies at our institution have shown that esophageal IMPT plans are generally robust to anatomical changes, and diaphragm amplitude variation was not a significant predictor of ITV coverage compared to the diaphragm baseline shift [34]. Consistent with this, our cohort (Fig. S1) showed high inter-fraction variability but decreasing median deviations over time. The number of breathing scenarios was also limited compared with what is recommended by Pastor-Serrano et al. to minimize statistical noise (more than 500) [29]. However, low RDE values and the excellent agreement between the *a posteriori* and predictive cumulative dose distributions support the adequacy of our reduced-scenario approach for clinical application. Finally, the predictive method currently relies on machine log files and therefore requires a pre-treatment dry run; integration of beam delivery time prediction into the treatment planning system may remove this requirement in the future [27].

In conclusion, the findings suggest that ITV-based robust optimization, conventional fractionation, and the Mevion S250i delivery characteristics provide sufficient robustness for most lung and esophageal cancer patients treated in free-breathing without rescanning. In contrast, studies using smaller spot-size systems often report the need for rescanning to mitigate interplay effects [8], [14], [31], [43].

However, small targets with large motion remain at risk and require careful patient-specific evaluation, particularly regarding beam-angle selection and motion-induced changes in target coverage. These conclusions are specific to the fractionation scheme studied here; hypofractionated treatments should be assessed separately.

## Declaration of generative AI and AI-assisted technologies in the manuscript preparation process

During the preparation of this work, the authors used Microsoft Copilot for textual improvements. After using this tool, the authors reviewed and edited the content as needed and take full responsibility for the content of the published article.

## CRediT authorship contribution statement

**Giorgio Cartechini:** Writing – review & editing, Writing – original draft, Visualization, Software, Project administration, Methodology, Investigation, Formal analysis, Data curation, Conceptualization. **Esther Kneepkens:** Writing – review & editing, Investigation. **Gloria Vilches-Freixas:** Writing – review & editing, Investigation. **Richard Canters:** Writing – review & editing, Investigation. **Mirko Unipan:** Writing – review & editing, Investigation. **Marije Velders:** Writing – review & editing, Investigation, Data curation. **Indra Lubken:** Data curation. **Maud Cobben:** Investigation, Data curation. **Anouk Vullings:** Investigation, Data curation. **Janou Buck:** Investigation, Data curation. **Maud van den Bosch:** Investigation, Data curation. **Steffy Paulissen:** Investigation, Data curation. **Julie Schoemakers:** Investigation, Data curation. **Dianne Hartgerink:** Investigation. **Judith van Loon:** Investigation. **Stephanie Peeters:** Writing – review & editing, Investigation. **Ilaria Rinaldi:** Writing – review & editing, Writing – original draft, Visualization, Validation, Supervision, Resources, Project administration, Methodology, Investigation, Data curation, Conceptualization.

## Ethics declaration

Written informed consent to take part in the study and to publish the article has been obtained from all participants or their legal representatives. The privacy rights of participants have been observed.

This study was performed in compliance with relevant laws, regulatory frameworks and guidelines where the research took place. This study was approved by the Maastro Institutional Review Board. (Approval No. P0719)

## Declaration of competing interest

The authors declare that they have no known competing financial interests or personal relationships that could have appeared to influence the work reported in this paper.
