# Supplementary material for: Interplay effect evaluation on lung and esophageal cancer patients treated with compact proton system in free breathing
Source: Phys Imaging Radiat Oncol. 2026 Sep 17;41:101085. doi: 10.1016/j.phro.2026.101085 (PMC13627791; doi:10.1016/j.phro.2026.101085)
Supplement: Supplementary file 1 [file mmc1.pdf]

A Supplementary Materials

A.1 Esophageal cancer patients' diaphragm amplitude variation

In Figure S1, we plotted the diaphragm amplitude of the 18 esophageal patients as a function of the repeated CT scans acquired during the treatment course. The first planning CT is assigned a CT number of zero. The diaphragm amplitude is calculated based on the displacement of the lower lobe of the lung across the 4DCT phases, with the maximum value between the left and right lungs being considered. The lower lobe is defined as the lowest point in the superior-inferior direction at the midpoint of the lung.

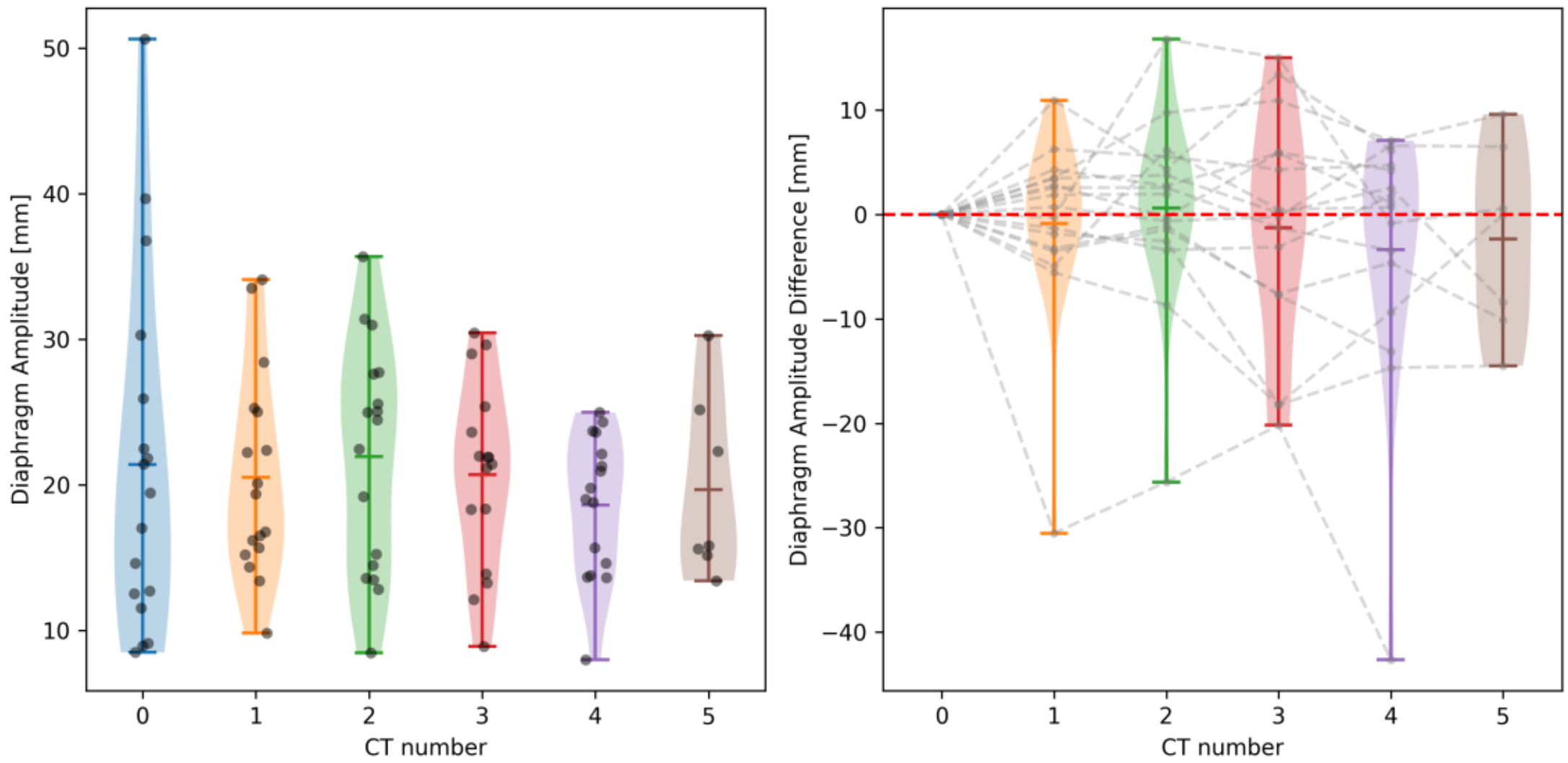

Figure S 1. The left panel shows the distribution of the diaphragm amplitude as a function of the periodic repeated CT over the treatment for 18 patients. On the right panel is shown the variation in the amplitude with respect to the first planning CT as a function of the repeated CTs. The gray dashed line connects the data of the same patient.

A.2 Esophageal patient 015

Figure S2 shows the beam arrangement between the A- and B-plans of the esophageal patient who failed the interplay and 4D evaluation. While the A-plan used three coplanar beam angles (150, 180 and 210 degrees), the B-plan is characterized by one oblique posterior-anterior beam, one posterior-anterior beam and one posterior-anterior tilted beam [1].

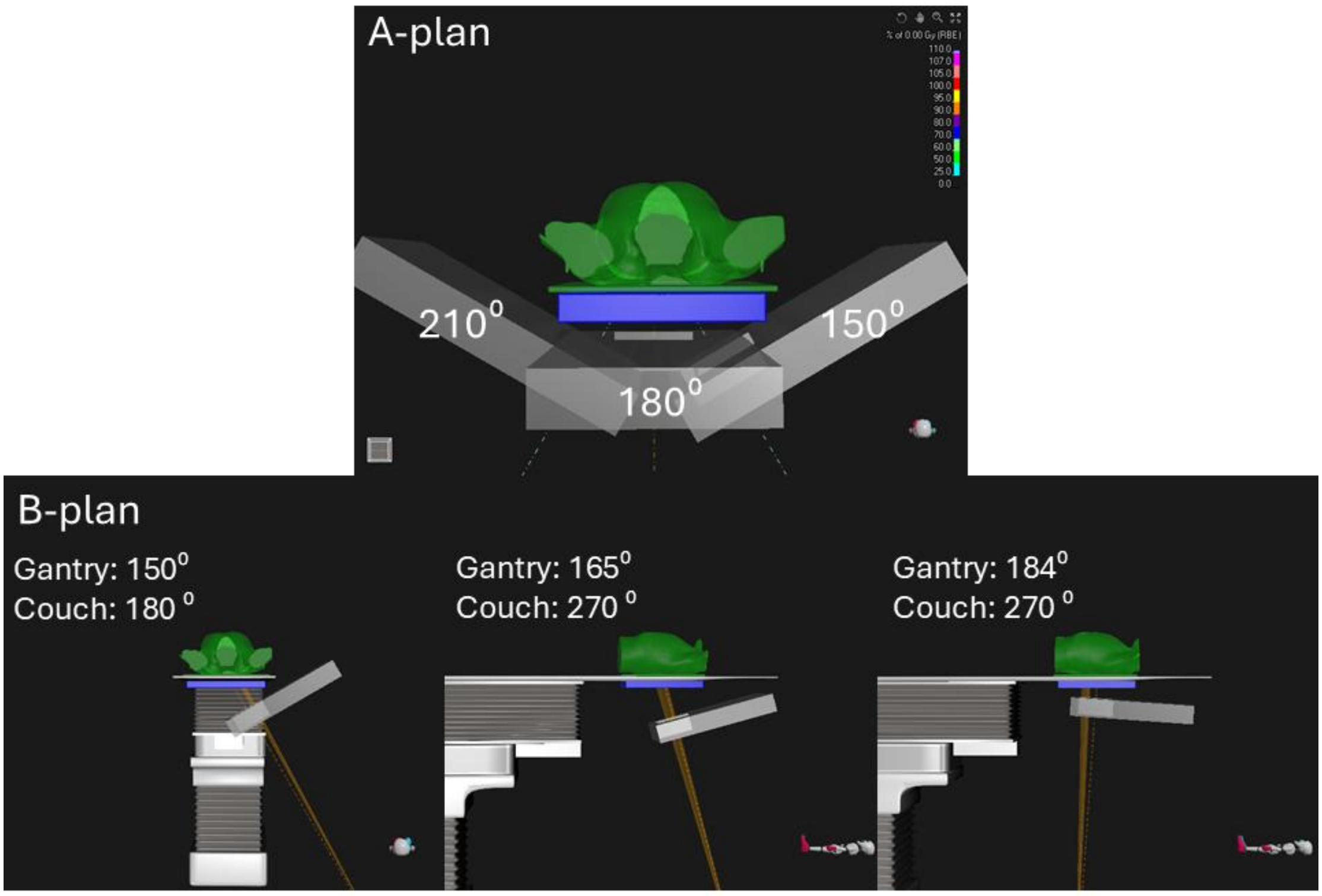

Figure S 2 Schematic representation of the A-plan and B-plan beam arrangement.

A.3 4D robust evaluation

The following tables report the values of the VWmin dose distribution generated by the 4D evaluation developed at our institution. For lung patients, we reported the CTVp D95 and 95% of the prescribed dose (Dp), while for esophageal cancer patients, we reported the V94 of the CTVp. To pass the evaluation, the D95 must exceed 95% of the D_p (lung) or the V94 must be larger than 98% of the CTVp volume (esophageal).

Tables

Table S 1 Summary of the main lung cancer patients' characteristics as well as the 4DRE parameters. To pass the 4D evaluation, the VWmin D95 must exceed 95% of the prescribed dose (D_p) on the CTVp and the VWmin V95 ≥95% of the CTVp volume. The cell highlighted in light red shows the plan that failed the evaluation.

ID	Plan	CTV Volume [cm³]	GTV Amplitude [mm]	4DRE VWmin D95 [Gy] ≥ 95% D_p	95% D_p [Gy]	4DRE VWmin V95 [%] ≥95%
**L1**	A	87	9.7	44.45	42.75	99.47
**L2**	A	81	5.1	58.31	57.00	99.17
**L2**	B	75	13.0	58.20	57.00	98.13
**L3**	A	155	9.3	53.36	52.25	98.52
**L3**	B	148	10.9	53.88	52.25	99.19
**L4**	A	176	7.2	54.18	52.25	99.70
**L4**	B	118	7.2	54.08	52.25	99.50
**L5**	A	151	6.4	59.16	57.00	99.80
**L6**	A	33	9.0	53.92	52.25	99.88
**L7**	A	115	5.1	53.85	52.25	98.71
**L8**	A	16	10.7	51.27	52.25	94.58
**L9**	A	262	22.8	58.68	57.00	98.77
**L10**	A	262	5.0	58.81	57.00	99.49
**L11**	A	239	7.2	58.99	57.00	99.78
**L12**	A	50	5.1	58.22	57.00	98.97
**L13**	A	202	6.6	58.46	57.00	99.55
**L14**	A	223	14.9	57.93	57.00	98.05
**L15**	A	82	5.1	53.75	52.25	98.36
**L16**	A	58	12.3	54.15	52.25	98.54
**L17**	A	152	9.7	54.02	52.25	99.73
**L18**	A	242	12.6	58.98	57.00	99.32

Table S 2 Summary of the main esophageal cancer patients' characteristics as well as the 4DRE parameters. To pass the 4D evaluation, the VWmin V94 must exceed 98% of the CTVp volume. The cell highlighted in light red shows the plan that failed the evaluation.

ID	Plan	CTV Volume [cm³]	Diaphragm Amplitude [mm]	4DRE V94 VWmin [%] ≥ 98%
**O1**	A	291	8.5	99.3
**O2**	A	232	8.9	99.7
**O2**	B	213	13.2	98.4
**O3**	A	181	22.5	98.5
**O4**	A	281	14.6	99.2
**O5**	A	489	11.5	99.5
**O5**	B	500	13.4	98.3
**O6**	A	219	9.1	99.4
**O6**	B	197	15.1	99.5
**O7**	A	156	20.1	99.7

**O7**	B	163	50.6	99.3
**O8**	A	179	30.3	99.4
**O8**	B	174	20.9	99.3
**O9**	A	99.7	19.4	99.5
**O10**	A	150	25.9	99.8
**O11**	A	508	36.8	99.8
**O12**	A	140	17.0	98.9
**O13**	A	199	21.8	98.8
**O14**	A	259	12.5	99.2
**O15**	A	58	39.7	93.5
**O15**	B	58	39.7	98.9
**O16**	A	510	21.4	99.7
**O17**	A	263	9.1	99.7
**O18**	A	527	12.7	98.9

A.4 Interplay evaluation on lung cancer patients with GTV amplitude larger than 2 cm

One of the current exclusion criteria for treating lung cancer patients with proton therapy in our center is a GTV motion amplitude larger than 2 cm. We selected three patients previously treated at our institution with conventional photon radiotherapy and generated corresponding proton therapy plans following the clinical protocol. Interplay analysis and four-dimensional robustness evaluation (4DRE) were then performed to assess the robustness of each plan. Table S3 summarizes the patient characteristics together with the 4DRE metrics. Figure S3 reports the relative differences between the cumulative D4DD dose metrics and those of the nominal plan.

Among the three cases, P1 demonstrated the highest level of robustness, with the cumulative dose converging toward the nominal plan after only a few fractions. In contrast, P2 and P3 exhibited the largest dose variations during the initial fractions; these deviations were partially mitigated by fractionation. Nevertheless, both patients presented a persistent systematic underdosage of approximately −4% for D98 and −2% for D95 and V95 at the end of treatment. These findings are consistent with the 4DRE results: while the VWmin D95 and V95 values for P1 remained well above the clinical acceptance thresholds, the corresponding values for P2 and P3 were close to the lower limits, indicating reduced motion robustness in these patients.

Table S 3Summary of the three photon lung cancer patients' characteristics as well as the 4DRE parameters. To pass the 4D evaluation, the VWmin D95 must exceed 95% of the prescribed dose on the CTVp. P1 was planned with three posterior beams, while P2 and P3 were planned with 2 'Twin beams' (Tb) posterior beams.

ID	Fields	Fractionation [Gy x Fx]	CTV Volume [cm^3]	GTV Amplitude [mm]	4DRE D95 VWmin [Gy]	95% of D_p [Gy]	4DRE V95 VWmin [%]
**P1**	3	2.75 x 20	8.4	24.5	54.86	52.25	100

**P2**	2 Tb		103.4	28.1	52.68	52.25	96.19
**P3**	2 Tb		214.5	20.3	52.51	52.25	95.39

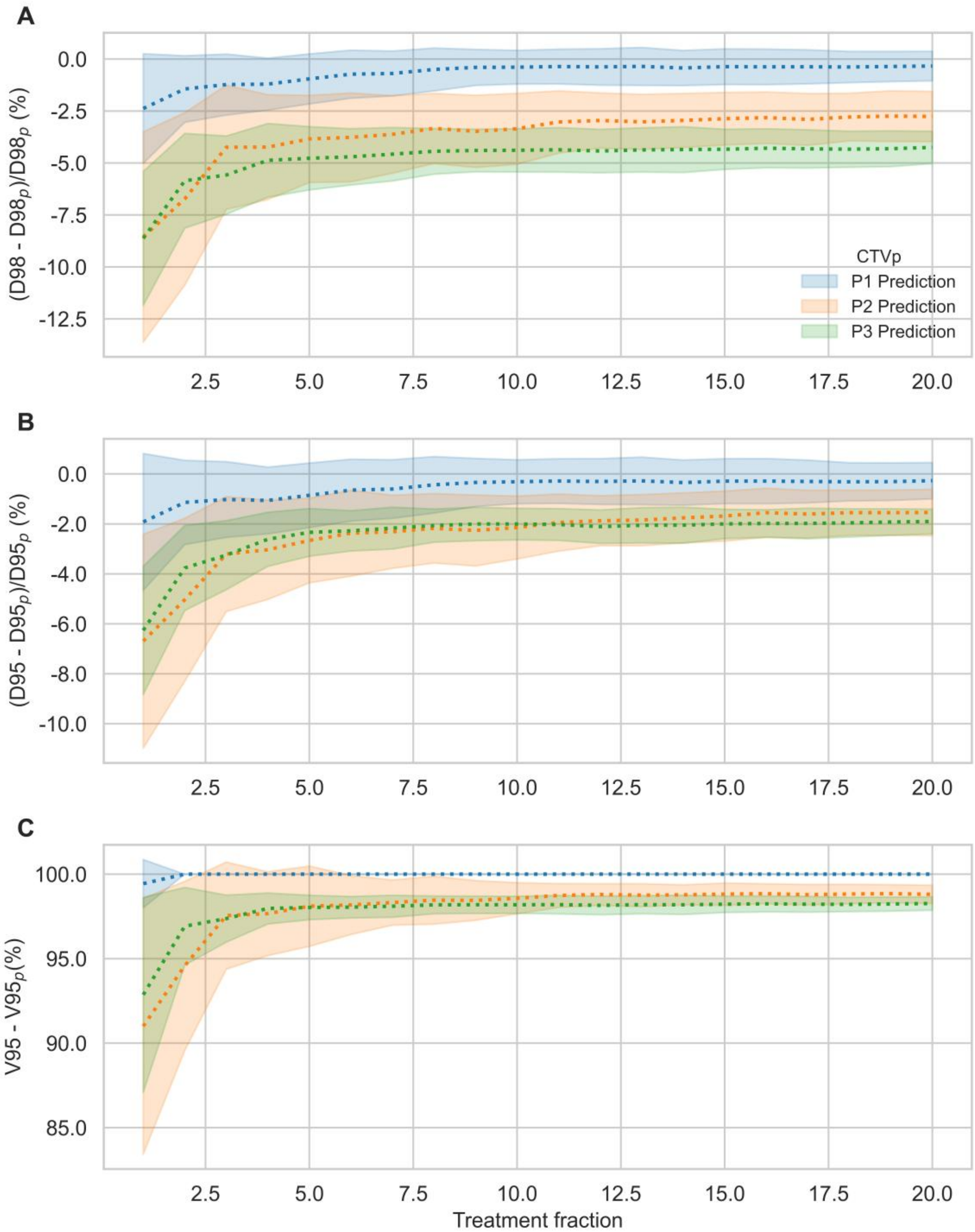

Figure S 3 Panels display the relative difference between accumulated D4DD D98, D95, and V95, and the planning values. The color band represents the standard deviation of the predictive method computed over synthetic treatment courses, while the dashed line represents the average value.

References

[1] Vilches-Freixas G, van der Klugt K, de Laat K, Buijsen J, Canters R, Berbee M. 2609: Towards a more robust proton beam arrangement against diaphragm changes for esophageal cancer. Radiotherapy and Oncology 2024;194:S2256–9. https://doi.org/10.1016/S0167-8140(24)02784-1.
